# Asymmetry in patient-related information disrupts pre-anesthetic patient briefing

**DOI:** 10.1186/1471-2253-13-29

**Published:** 2013-10-04

**Authors:** Joerg Schnoor, Anja Kupfer, Babette Jurack, Ulrike Reuter, Herrmann Wrigge, Steffen Friese, Volker Thieme

**Affiliations:** 1 Department of Anesthesiology and Intensive Care, University Hospital Leipzig, Liebigstr. 20, Leipzig 04103, Germany

**Keywords:** Information asymmetry, Pre-anesthetic patient briefing, Disruptions, Time delay, Personnel costs

## Abstract

**Background:**

If one party has more or better information than the other, an information asymmetry can be assumed. The aim of the study was to identify the origin of incomplete patient-related preoperative information, which led to disruptions and losses of time during pre-anaesthetic patient briefing. We hypothesized that lower employees’ educational level increases the amount of disruptive factors.

**Methods:**

A prospective observational study design was used. Patient selection was depending on the current patient flow in the area of the clinic for pre-anesthetic patient briefing. Data were collected over a period of 8 weeks. A stopwatch was used to record the time of disruptive factors. Various causes of time losses were grouped to facilitate statistical evaluation, which was performed by using the U-test of Mann and Whitney, Chi-square test or the Welch-t-test, as required.

**Results:**

Out of 221 patients, 130 patient briefings (58.8%) had been disrupted. Residents were affected more often than consultants (66% vs. 47%, p = 0.008). Duration of disruptions was independent of the level of training and lasted about 2,5 minutes and 10% of the total time of patient briefing. Most time-consuming disruptive factors were missing study results, incomplete case histories, and limited patient compliance.

**Conclusions:**

Disruptions during pre-anesthetic patient briefings that were caused by patient-related information asymmetry are common and account for a significant loss of time. The resultant costs justify investments in appropriate personnel allocation.

## Background

In Germany, after implementation of a system of diagnosis-related groups (G-DRG), anecdotal evidence has shown that the provision of medical information has become increasingly difficult. A growing lack of pre op medical findings in the anesthetic room necessitates increased efforts by anesthetic staff for compensation. If one party has more or better information than the other, an asymmetry of information can be assumed. In case of incomplete patient-related information, asymmetry of information can be generated in an intended or unintended manner.

Approximately 60% of overall costs in German hospitals are staffing costs [1]. The corollary during the last decade was thus an attempt to streamline the human resources sector with a simultaneous rejuvenation. Economically justified savings in the human resources sector, however, had a significant potential to hamper well organized and even proven process chains. Within clinical process chains, a pre-anesthetic clinic for patient assessment and briefing represents an essential cutting point.

Patients’ past medical history, medication, and physical examination merge into a preoperative image of the patient’s overall status, mandatory to choose the safest method of anesthesia. To meet this clinical standard, essential requirements for pre-anesthetic patient education include diverse information about patients’ past medical history, medication, examination findings, and the precise knowledge about planned surgical procedures. These inputs usually form the basis for a sufficient patient briefing, which takes place in a “front-office area” exerting profound influence on patient satisfaction and, probably, treatment outcome [2,3].

To date, there are limited data considering the aspects of incomplete information during pre-anesthetic patient assessment and briefing. The aim of this study was to identify the origin of this particular loss of information, which led to disruptions and loss of time during pre-anesthetic patient assessment and briefing. Therefore, disruptive factors were prospectively recorded and compared in relationship to the employees’ educational levels. We hypothesized that the lower an employees’ educational level, the higher the impact of the amount of disruptive factors.

## Methods

The study was approved by the local ethics committee (Ethik-Kommission der Medizinischen Fakultät der Universität Leipzig, Ref:176-11-30052011). Written informed consent was obtained from all subjects. Adults with ASA-classification I-IV were allowed to be enrolled in this study [4]. Criteria for exclusion were children and patients under the age of 18 years, missing surgical consent forms, inadequate language skills, previous participation in the study, and patient’s refusal to participate.

### Process flow analysis

A process flow analysis was used to identify both the core and the subsidiary process of inpatient surgical patient care. The original value-adding process for obtaining a lump sum payment defined the core process. All supportive measures were called subsidiary processes.

### Open problem description of disruptions

An open problem description was used to identify all disruptive factors by local employees in a subjectively manner. A disruptive factor was defined as a time-measurable event that interrupts a continuous pre-anesthetic patient assessment and briefing or generates additional and unintended waiting periods, or required supplementary measures for staff.

### Real-time detection of disruptions

Additional staff performed prospective recordings of all disruptions parallel to the pre-anesthetic consultation and briefing. All participants were informed about the content of the examination. Patient selection was depending on the current patient flow in the area of the clinic for pre-anesthetic patient assessment. Data was collected over a period of 8 weeks during the summer holidays. For documentation a specially designed documentation sheet was used (see Additional file 1). A stopwatch was used to record the time of disruptive factors. Various causes of time loss were grouped to facilitate statistical evaluation.

### Staffing costs

Measured times of disruptive factors were then allocated to medical staffing costs for a local tariff. To consider both ancillary labor costs and overhead expenses, the resulting medical staffing costs were calculated as 56.44 £/hour (70 € and 90.30 $ per hour, respectively) for consultants and 40.31 £/hour (50 € and 64.50 $ per hour, respectively) for residents.

### Analysis

After data collection, disruptive factors were transmitted to a spreadsheet program (Excel®, Windows XP). A parametric test (Welch two-sample t-test) was used to compare age groups, because data could be characterized as distributed normally (Kolmogorov-Smirnov test). In contrast, all further comparative statistics were made using the u-test according to Mann and Whitney as well as the chi-square test (Pearson’s correlation) for non-parametric data (SPSS® IBM® version 20). Data are given as mean (SD), median (interquartile range) or number and proportion, when required. The significance level was set at p <0.05.

## Results

### Process flow analysis

**Figure 1:**
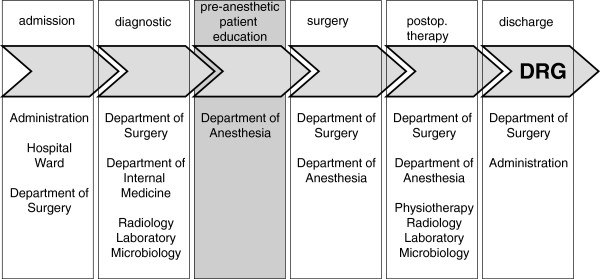
**Process flow analysis demonstrating both the core and the subsidiary process of inpatient surgical patient care.** The value-adding process for obtaining a lump sum payment (DRG) was defined the core process.

The identification of core and secondary processes are demonstrated by Figure 1. The value-adding core process is characterized by achievement of the lump-sum payment (DRG) by means of surgery. The anesthetic preoperative patient assessment and briefing is one of the six subsidiary processes of a surgical treatment.

### Open problem description of factors of disruptions

**Figure 2:**
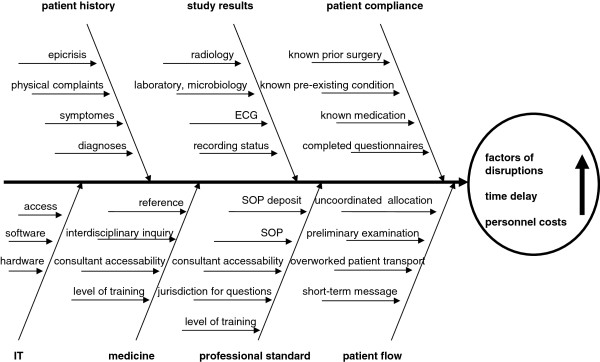
Ishikawa diagram demonstrating possible causes of disruptive factors in the area of a clinic for pre-anesthetic patient briefing.

From an anesthetic perspective, the employee survey (n = 8) revealed various disruptive factors in a decreasing order of frequency (Table 1). Most often, the desire for complete set of patient records, past medical history and information about operational procedures was stated. Depending on level of training (consultants vs. residents) documentation of the measured confounders yielded different causes for disruptions (Table 2). From this, seven different groups of disruptions (patient history, medical findings, professional standards, medicine, patient flow, patient compliance, and information technology (IT)) were identified. A diagram of Ishikawa was used to visualize causalities (Figure 2).

**Table 1:** Employee’s survey (n = 8) to subjectively experienced causes of disruptions in a decreasing order of frequency

**Frequently named disruptive factors**	
•	Incomplete medical results or records
•	Lack of patient history
•	Long waiting time for patients caused by uncoordinated patient flow
•	Uninformed patient about own surgery
•	No regular surgical contact for requests
•	No visual aids for anesthesia
•	Time consuming IT system*

*IT information technology.

**Table 2:** Identified disruptive factors depending on the level of education (consultants vs. residents) and frequency

**Identified factors by consultants**	**Identified factors by residents**
PC-hardware problems*	IT medical research^†^
Lack of information about medication	Anaesthesiological contact for request
Lack of medical findings	IT laboratory request^†^
Lack of information about case conference	Incomplete patient questionnaire
Surgical contact for request	Lack of information about case conference
Anaesthesiological contact for request	Lack of patient history
	Lack of information about medication
	Missing surgical contact for request
	Lack of allergy passes
	PC-hardware problems*
	PC-software problems*

*PC personnel computer, ^†^*IT* information technology.

### Real-time detection of disturbances

In total, 221 patients were monitored and logged. The statistical group comparison of biometric data is demonstrated in Table 3. Except for age, normality tests demonstrated data to be non-parametric (p < 0.02). Patient’s age showed significant differences between the groups with older patients who had been visited by consultants (p = 0.018). Except for age, statistical significant differences could not be found between the groups (Table 3).

**Table 3:** Biometric data and time of patient briefing (consultants vs. residents)

	**Consultants**	**Residents**	**p**
	**(n = 79)**	**(n = 142)**	**value**
Age; years	58.7 (15.8)	52,6 (16.4)	0.018
ASA	II (I–III)	II (I–IV)	0.311
BMI	26,8 (22.8–29.4)	25,8 (23.0–29.1)	0.143
t-total-edu*; min	1 370	3 711	0.001
t-median-edu^†^; min	16.0 (12.0–21.0)	21.6 (16.0–29.1)	0.250

*t-total-edu; total time of pre-anesthetic patient briefing. ^†^t-median-edu; median time of pre-anesthetic briefing.

Except for age (mean (SD)), values are given as median (interquartile range) or number.

Residents (n = 28) visited 64% of all patients. Residents were between their first and fifth year of training, with a median of a second year of training. The median time of pre-anesthetic patient education (t-median-edu) was comparable between the groups. Data demonstrates a trend for shorter patient briefings when consultants (n = 33) were involved (Table 3).

Out of 221 pre-anesthetic patient briefings, 130 briefings (58.8%) suffered disruptions. Assessments and briefings by residents were more often disrupted than those by consultants (p = 0.008). The median time of disruption (t-median-disrupt) showed no significant differences between the groups (p = 0.396). The resulting proportion of median failure time at the median briefing time (t-part-disrupt = t-median-disrupt/t-median-edu) was comparable between the groups and lasted about 10% of the overall assessment time (Table 4).

**Table 4:** Time of disruptions (consultants vs. residents)

	**Consultants**	**Residents**	**p value**
Number of patients	37 (46.5%)	93 (65.5%)	0,008
t-total-disrupt*; min	127	391	
t-median-edu-disrupt^†^; min	17.9 (13.6–24.7)	24.5 (19.5–31.7)	0,534
t-median-disrupt^±^; min	2.3 (0.8–4.9)	2.5 (0.7–4.7)	0,396
t-part-disrupt^§^;%	12.8	10.2	

*t-total-disrupt; total time of disruptions. ^†^t-median-edu-disrupt; median time of pre-anesthetic patient education that has been disrupted; ^±^t-median-disrupt; median time of disruptions. ^§^t-part-disrupt; (t-median-disrupt * 100 /t-median-edu-disrupt).

Values are given as number, median (interquartile range), or proportion.

Each group of disruptions revealed a different time requirement (Table 5). For consultants, the most time-consuming causes of disruption were missing medical findings and incomplete case histories. Limited patient compliance and time loss because of the physicians’ use of the IT-system were other causes of disruptions. In total, these four groups of disruptions generated 94.5% of all causes when consultants were involved.

**Table 5:** Quantity of disruptive factors (number), duration (minutes) and proportion (%) during patient briefings (consultants vs. residents)

	**Consultants**			**Residents**			**p value**
	**Number**	**Min**	**%**	**Number**	**Min**	**%**	
Medical findings	21	46	36.2	47	225	57.5	0,007
Patient history	9	31	24.4	15	27	6.9	0.010
Patient compliance	11	27	21.3	17	43	11.0	0.020
IT*	10	16	12.6	45	71	18.2	0.759
Professional standards	2	7	5.5	16	19	4.9	0.803
Medicine	0	0	0	3	6	1.5	0.851
Total	53	127	100	143	391	100	

*IT information technology.

For residents, 57.5% of patient interviews showed disruptions caused by a lack of medical findings (Table 5). A frequent reason for disruption was the time-consuming use of the IT-system. A decrease in patient compliance caused further failure time. An incomplete patient history and the need for information about professional standards or medical background information (medicine) were minor causes.

### Staffing costs

In 130 cases of disrupted patient briefings, the time of disruptions combined amounted for more than 8 hours while the average time delay was 2.5 minutes. When disrupted, each patient briefing by consultants generated additional costs of £ 2.26 (€ 2.80 and $ 3.61, respectively) on average. When residents saw patients, disrupted patient assessment and briefings generated additional costs of £ 1.68 (€ 2.08 and $ 2.68, respectively) per patient on average.

At a hospital with 25 000 anesthetic patient assessments per year, this would amount to about 122 additional working days to compensate for the delay caused by these disruptions alone. In case of pre-anesthetic patient assessment performed by residents, additional staffing costs of £ 39 553 (€ 48 831 and $ 62 992, respectively) per year should be expected for compensation. Alternatively, compensating for time delays for consultants would cost £ 55 374 (€ 68 363 and $ 88 188, respectively) per year.

## Discussion

Data demonstrates that more than every second anesthetic patient assessment had been disrupted. Residents were more often affected than consultants. The resulting delay was independent of the level of training and lasted about 10% of the total briefing time. Depending on the level of employee’s level of education, disruptive factors generated additional costs between £ 1.68 and 2.26 per patient.

Interdependent of the level of education, most time consuming disruptive factors were the lack of medical findings. Rather than missing results of more technical investigations (echocardiography, chest X-ray, hematologic laboratory), we also found an imperfection of simpler physical examination results like blood pressure, body weight, and body height (data not shown) that were usually provided by nurses. While pre-anesthetic anticipation and planning are essential steps of risk management, missing test results and incomplete transfer of information can promote erroneous conclusions leading to poor decisions that ultimately jeopardize patient safety [5]. Also, life-threatening medication errors can be the result of missing or illegible documentation [6]. This is of rising concern since stakeholders in health care clearly emphasize particular interests in quality and safety of patient care. Conclusively, there still might be a contradiction between stated objectives and actual outcome that is simply caused by incomplete data collection. This might be due to cost-cutting measures through the change from cost allowance to a system of lump sum payment (DRG) associated with politically motivated staff shortages within the health care sector. As of now, this has induced notable reductions in average nursing minutes per day [7].

In general, the DRG-system promotes concentration on core processes; meanwhile the allied health care/nursing sector is still mapped incomplete for financial revenues. In consequence, incomplete examination findings from the allied health care/nursing sector has to be completed by doctors, which generates both increased processing times and staffing costs skipped to just a different part of the medical sector, possibly followed by an increase of total costs.

The second most common disruptive factor for consultants was a missing past medical history. Normally, past medical history of patients is initially documented by the surgeon prior to indicating the planned procedure. Even in the medical field, budgeted revenues complicate the timely fashioned recoding of the past medical history. As a result, composing a complete past medical history has to be done by the anesthesiologist.

Residents were more affected by a time-consuming use of IT systems. With demographic changes there is a rising proportion of elderly patients suffering multiple comorbidities and polypharmacy. The fragmented nature of delivering health care, the large volume of transactions, the need to integrate scientific evidence into practice, and a rising transfer of information make electronic information management systems more appropriate to address continuous quality improvements and patient safety initiatives [8,9]. Here, IT is at the crossroads of technology and patient safety. Anesthesia Information Management Systems (AIMS) show the potential to improve patient safety and quality in the perioperative setting. AIMS make legibility of documentation more feasible, which, in consequence, helps optimizing the exchange of complex health information [8]. In contrast, our data shows that using computer-based documentation and information systems was a significant nuisance. Overall, it is surprising that while financial investments to set up IT solutions had been justified by forecasted timesaving, nevertheless we found that about 1/5 of the time of disruption was caused by its use. Disruptions by IT have already been described. Criticism includes discomfort with rapid documentation and electronic data entry during short or emergency procedures and inconvenient placement of the system at the anesthesia workstation. A further barrier to adoption and implementation of IT seems to be the question about legal status with missing or outlier data [8,10]. In total, IT adoption will be decreased when interfaces between different software manufacturers or the lack of active physician involvement in planning, designing, and implementation of IT solutions still impede clinical practicability [8]. At present, it remains open to question whether local solutions should be sought in optimized user training, in user-oriented software programming or in clinically appropriate hardware development. In this context, the extent to which the vision of complete paperless documentation can be realized with respect to cost-effectiveness remains to be seen.

For both consultants and residents the third most common disruptive factor was a restricted patient compliance through incomplete patient questionnaires. This was because most patients did not utilize their waiting time prior to the anesthetic assessment and briefing. In a few cases, patients were unable to complete the questionnaire by themselves. Here, nurses had no time to help with filling out questionnaires. As a result, the task was performed by the anesthesiologist, which took between 11 and 21% of the total fault time.

Both the taking of patient’s past medical history and the physical examination are the basis of individual risk stratification of patients. Therefore, pre-anesthetic assessments promote patient safety. Our data demonstrate that information asymmetry then residents less affected than consultants. In consequence, running an anesthetic pre-assessment clinic just by consultants might optimize patient contact time. However, the outcome of such a consultant- only approach of pre-anesthetic assessment remains to be seen. Although preoperative patient information alone might have no influence on the recuperating process, there is some evidence that preoperative relief of patient anxiety optimizes postoperative recovery and shortens hospital stays [11-14].

Clinics for pre-anesthetic patient consultation allow for a faster patient care through economies of scale and saving spatial mobility with shorter set-up time on the side of staff. In general, high patient turnover places demands on concentration, organizational and communication skills, and social competence of staff. This activity in the residential sector is not redemptive or relevant. Staff costs for pre-anesthetic patient consultation and briefing had been estimated with 6% of the total staffing costs [11,12]. It is charged as internal DRG revenue. Since around 60% of fixed costs in German hospitals were staffing costs, the last decade has seen a unique corollary attempting to streamline human resources as well as their rejuvenation [1]. Subsequently, the quantity of disrupted patient briefings increased when residents were involved. Additionally, the time needed for patient consultations increased as well. The average time for a patient’s evaluation is estimated at 20 minutes, which was missed on average by residents. An additional colleague for help or training is not provided by the DRG-system. Each type of disruption may hinder the process of patient briefing with resulting costs exceeding the income, particularly, when repeated processes and glitches add a significant expense. Also, disruption-related impairment of employee’s power of concentration favor omissions and, possibly, wrong decisions. All these factors might negatively influence the quality of results. Conclusively, defined and consented standard procedures promise beneficial effects on process design, development costs, and quality.

Therefore, simple interdisciplinary arrangements to reduce asymmetries in information have been taken: first, prior to the patient’s presentation, simple test results (e.g. blood pressure, body weight and others) have to be documented by the caregivers. In addition, complete information regarding past medical history, medication, and the planned operational measures must be available. The data completeness has to be checked in the field of the anesthesia clinic right before consultation. Secondly, patients have to complete their questionnaire during their waiting period that has to be check by a nurse. These measures are mandatory prerequisites before patient consultation by the anesthesiologist. It seems advisable to implement these simple measures accordingly, although patient status should allow exceptions. However, if exceptions reign supreme, this will be a none sustainable solution to the problem of asymmetry of information.

### Limitations of data

The study has some limitations. Time delays caused by uncoordinated patient flow were not detected due to the structure of the clinical patient flow, since patients were present at the actual briefing time, because they had been waiting in an office area. This functioned as a bottleneck right before the doctor’s consultation by generating continuous patient flow. All patients enrolled were mobile and came to the clinic for anesthetic pre-assessment and briefing on their own. We did not include any immobile or bedbound patients. These patients might have prolonged consultation times mostly caused by increased morbidity associated with medical records that took longer to survey. This group of patients has a high potential for interferences as well as higher staffing costs.

A bias might be caused by a lack of randomization. With regard to the medical level of employees training, patient’s assignment has to be assumed with consultants visiting the older patients. According to the usual routine, patients with complex medical conditions could have been assigned to the more specialized anesthesiologist although we did not observe differences in ASA classes between groups. Also, the selection of accompanied patients had been carried out in availability of an additional person for documentation, which might have influenced the sample being investigated. Therefore, randomization might have more clearly shown that briefing times by consultants were shorter than those by residents.

Grouping the different causes of faults was based on documentation. Each decision to assign the cause of disruption to each group was performed in a subjective manner and therefore might be biased. Finally, treatment outcome and patient satisfaction were not considered. Thus, it remains unclear whether and to what extent disruptive factors affect the quality of outcome.

## Conclusions

Our findings demonstrate the nature and extent of disruptions caused by incomplete information during pre-anesthetic patient assessment and briefing. Residents were more often affected than consultants, and the resulting delay per patient was independent of the level of training. The ensuing costs justify investments in both consented procedural orders and appropriate staff allocation.

## Abbreviations

ASA: American society of anesthesiologists; DRG: Diagnosis related groups; IT: Information technology; PC: Personnel computer; BMI: Body mass index; OMS: Oral and maxillofacial surgery; SOP: Standard operating procedures.

## Competing interests

The authors declare that they have no competing interests.

## Authors’ contributions

JS made substantial contributions to the conception, design, interpretation of the collected data, and drafted the manuscript. AK, BJ and UR performed the study and provided critical review of the manuscript. HW, SF and VT contributed to the interpretation of the data and provided critical review of the manuscript. All authors read and approved the manuscript.

## Pre-publication history

The pre-publication history for this paper can be accessed here:

http://www.biomedcentral.com/1471-2253/13/29/prepub

## Supplementary Material

Additional file 1 Schedule of disruptions. Click here for file
