# Supplementary material for: Asymmetry in patient-related information disrupts pre-anesthetic patient briefing
Source: BMC Anesthesiol. 2013 Oct 4;13:29. doi: 10.1186/1471-2253-13-29 (PMC3851798; doi:10.1186/1471-2253-13-29)
Supplement: Additional file 1 — Schedule of disruptions. [file 1471-2253-13-29-S1.pdf]

schedule of disruptions

Date:……………………
Day: ◻ Monday ◻ Tuesday ◻ Wednesday ◻ Thursday ◻ Friday

Start of consultation: ____________h

Patient data:

gender: ◻ female ◻ male
age: ……..…… years
body height: …………… cm
body weight: …………… kg BMI………

ASA: ◻ 1 ◻ 2 ◻ 3 ◻ 4

Surgery:

◻ Dermatology	◻ vascular surgery	◻ Gynecology	◻ Otolaryngology	◻ pediatric surgery	◻ OMS
◻ Neurosurgery	◻ ophthalmic surgery	◻ orthopedics	◻ Traumatology	◻ Urology	◻ visceral surgery

Intervention: ………………………………………………………………………………………………

Consultation by : ◻ consultant ◻ resident

OMS = Oral and Maxillofacial Surgery ; SOP = standard operating procedure

area	aspects	status	transaction	Time loss (min)
**medical history:**	anesthesia questionnaire:	□ filled out already<br>□ not filled out		
	□ medical history fulfilled<br>□ anesthetic aspects of medical history	□ former anesthesia<br>□ allergy<br>□ medication<br>□ pre-existing conditions/disease:<br>□ cardiovascular risk<br>□ lung disease<br>□ gastric reflux<br>□ liver insufficiency<br>□ renal insufficiency<br>□ congenital muscle disease	□ telephone inquiries<br><br>□ internet inquiries	
**medical findings**	□ completely<br>□ request necessary	□ laboratory<br>□ ECG<br>□ cardiac echography<br>□ X-ray<br>□ others	□ telephone inquiries<br><br>□ internet inquiries	
	□ reevaluation necessary<br>□ postponed		□ telephone inquiries	
**patient information**		□ patient ready for consultation<br>□ patient not ready for consultation (forgotten glasses, anxiety, others)	□ delay in consultation	
	□ health record completely<br>□ health record not available<br>□ medical results present<br>□ medical results to organize	□ blood pressure<br>□ heart rate<br>□ body temperature<br>□ glucose<br>□ ability to open the mouth (cm)<br>□ auscultation<br>□ Mallampati score<br>□ dental status<br>□ neck mobility	□ telephone inquiries<br><br>□ internet inquiries	

**surgery**	□ known procedure:<br><br>□ known time of surgery	If yes, which kind of surgery?<br>______________________________<br>If yes, what time?<br>______________________________	□ telephone inquiries	
**anesthesia**	Anesthesia technique	□ general anesthesia<br>□ spinal/epidural anesthesia<br>□ regional anesthesia/nerve blocks<br>□ blood transfusion	□ telephone inquiries	
	monitoring:	□ standard<br>□ invasive<br>□ admission ICU	□ telephone inquiries<br><br>□ internet inquiries<br><br>□ application organized by telephone	
		□ pain therapy (catheter)	□ telephone inquiries<br><br>□ application organized by telephone	
		□ others<br>□ SOP Info	□ telephone inquiries<br><br>□ internet inquiries	
				total

End of consultation:…………….….……..h

Total time of consultation: ………………......min
